# A promising natural killer cell-based model and a nomogram for the prognostic prediction of clear-cell renal cell carcinoma

**DOI:** 10.1186/s40001-024-01659-0

**Published:** 2024-01-24

**Authors:** Qinfan Yao, Xiuyuan Zhang, Yucheng Wang, Cuili Wang, Jianghua Chen, Dajin Chen

**Affiliations:** 1 Kidney Disease Center, the First Affiliated Hospital, College of Medicine, Zhejiang University, Qingchun Road 79, Hangzhou, 310003 China; 2 Key Laboratory of Kidney Disease Prevention and Control Technology, Hangzhou, Zhejiang Province China; 3 Institute of Nephropathy, Zhejiang University, Hangzhou, China; 4 Zhejiang Clinical Research Center of Kidney and Urinary System Disease, Hangzhou, China

**Keywords:** Clear-cell renal cell carcinoma, Natural killer cells, TCGA, Risk score, Prognosis

## Abstract

**Background:**

Clear-cell renal cell carcinoma (ccRCC) is one of prevalent kidney malignancies with an unfavorable prognosis. There is a need for a robust model to predict ccRCC patient survival and guide treatment decisions.

**Methods:**

RNA-seq data and clinical information of ccRCC were obtained from the TCGA and ICGC databases. Expression profiles of genes related to natural killer (NK) cells were collected from the Immunology Database and Analysis Portal database. Key NK cell-related genes were identified using consensus clustering algorithms to classify patients into distinct clusters. A NK cell-related risk model was then developed using Least Absolute Shrinkage and Selection Operator (LASSO) Cox regression to predict ccRCC patient prognosis. The relationship between the NK cell-related risk score and overall survival, clinical features, tumor immune characteristics, as well as response to commonly used immunotherapies and chemotherapy, was explored. Finally, the NK cell-related risk score was validated using decision tree and nomogram analyses.

**Results:**

ccRCC patients were stratified into 3 molecular clusters based on expression of NK cell-related genes. Significant differences were observed among the clusters in terms of prognosis, clinical characteristics, immune infiltration, and therapeutic response. Furthermore, six NK cell-related genes (DPYSL3, SLPI, SLC44A4, ZNF521, LIMCH1, and AHR) were identified to construct a prognostic model for ccRCC prediction. The high-risk group exhibited poor survival outcomes, lower immune cell infiltration, and decreased sensitivity to conventional chemotherapies and immunotherapies. Importantly, the quantitative real-time polymerase chain reaction (qRT-PCR) confirmed significantly high DPYSL3 expression and low SLC44A4 expression in ACHN cells. Finally, the decision tree and nomogram consistently show the dramatic prediction performance of the risk score on the survival outcome of the ccRCC patients.

**Conclusions:**

The six-gene model based on NK cell-related gene expression was validated and found to accurately mirror immune microenvironment and predict clinical outcomes, contributing to enhanced risk stratification and therapy response for ccRCC patients.

**Supplementary Information:**

The online version contains supplementary material available at 10.1186/s40001-024-01659-0.

## Introduction

Clear-cell renal cell carcinoma (ccRCC) represents the most prevalent histological subtype of renal cell carcinoma (RCC), accounting for over 70% of the cases worldwide [1–5]. With its high invasiveness and recurrence, ccRCC annually causes more than 175,000 mortality rates worldwide and a poor 5-year survival probability of approximately 50% [6–10]. Although partial or radical nephrectomy is currently the optimal choice for ccRCC, about 25% of patients present with lymph node or distant metastasis at the first diagnosis and are unable to undergo surgery, while over 30% of ccRCC patients experience relapse and metastases after surgery [11–13]. The considerable exploration of prognostic models of ccRCC and the wide application of immune checkpoint-blocking therapies have gradually shown powerful potential and may offer new therapeutic options for ccRCC patients [14–18]. Emerging evidence has proven that immune cells serve an essential function in ccRCC pathogenesis, providing a novel outlook into immunotherapy for ccRCC [19–22]. The previously constructed immune/stromal scores have indicated a strong association between ccRCC prognosis monitoring and precision immunotherapy [23]. And the 3 immune infiltration patterns of ccRCC showed that ccRCC had the highest immune infiltration and T cell infiltration score [24]. Importantly, the complete single-cell chromatin chart of immune cells in ccRCC assisted us in better understanding immune cell functional states in ccRCC [25]. Meanwhile, several single-cell sequencing analyses provided an in-depth knowledge of the immune landscape and immune cell infiltration patterns of ccRCC, which contributed to setting a foundation for the theoretical basis of targeted therapy [26–28].

Recently, natural killer (NK) cells have been shown to play a critical role in immune surveillance against viruses, bacterial infections, and tumors [29–31]. NK cells encode both activating and inhibitory receptors and further integrate diverse signaling pathways to exert corresponding functional outcomes, especially in the production of cytokines and chemokines [32–34]. The significant advances in NK-cell biology and the gradual unveiling of molecular mechanisms have provided novel strategies involving the regulation of NK activation and cancer cell recognition, which are considered as important for cancer immunotherapy [35–39]. Moreover, immune cell-based prognostic models have been widely explored in clinical settings for guiding cancer management [40–42]. An increasing number of NK cell-related genes are being widely studied for their important roles in various types of tumors. For example, NKG2D, NKp30, and NKp46 trigger cytotoxic effects by binding to ligands on the surface of tumor cells, thus exerting anti-tumor effects [43–45]. And NK cells are recognized as a significant prognostic factor for multiple cancers. In gastric cancer, researchers have found that abnormal percentage of NK cell in peripheral blood predicts patients' poor survival rates [46]. In triple-negative breast cancer, a 5-NK cell-related gene model also exhibit powerful value for prognosis prediction and immunotherapy evaluation [47]. However, there is currently no comprehensive understanding on the role of NK cell-related genes in ccRCC.

Therefore, the objective of this study is to investigate the role of NK cells in the carcinogenesis process of ccRCC, and to evaluate the potential value of targeting NK cell-related genes for optimizing risk stratification and predicting treatment efficacy in ccRCC. We estimated associations between the expression features and functional features of NK cell-related genes with ccRCC, and constructed a prognostic risk model to estimate the tumor immune microenvironment, predict prognosis, explore the treatment response, and allow for risk stratification. Our findings can aid in accurate therapeutic decisions being made for ccRCC patients.

## Materials and methods

### Data collection and processing

The transcriptome RNA-sequencing (RNA-seq) data and relevant clinical information on ccRCC were retrieved from TCGA-KIRC project of the Cancer Genome Atlas (TCGA) database (https://portal.gdc.cancer.gov/projects/TCGA-KIRC). Total 134 genes associated with natural killer cells were obtained from the Immunology Database and Analysis Portal (ImmPort) database (https://www.immport.org/home) [48]. The RECA-EU database of the International Cancer Genome Consortium (ICGC) database was searched to obtain the corresponding RNA-seq data and clinical information (https://dcc.icgc.org/projects/RECA-EU) as validation set.

### Cell culture and qRT-PCR analysis

Human ccRCC ACHN cells and kidney proximal tubular epithelial (HK2) cells were cultivated in Dulbecco's modified Eagle’s medium (DMEM) with 10% fetal bovine serum (FBS) and 1% penicillin–streptomycin. All cells were cultured under humidified conditions at 5% CO_2_ and at 37 °C. We conducted a qRT-PCR to quantify the expression of the genes selected to be used in the model. Total RNA was obtained from the cultured cells using TRIzol reagent (Invitrogen, CA, USA). The PrimeScript™ RT reagent kit (Takara, Shiga, Japan) was applied to reverse transcribe the RNA into complementary DNA (cDNA), following the manufacturer’s instructions [49]. Sequences of all primers used are presented in Additional file 1: Table S1. The experiments on each gene were performed in triplicate and the average cycle threshold (Ct) was computed. The mRNA levels were normalized to that of the housekeeping gene, GAPDH, using the 2^−ΔΔCt^ method.

### Identification of NK cell-related genes specific to ccRCC prognosis and the establishment of a molecular cluster

A univariate Cox regression model analysis was performed on the TCGA database to select prognostic genes among the NK cell-related genes using the R package survival “coxph” function. Then, the R package, ‘ConsensusClusterPlus’, was used to construct a consistency matrix and classify the ccRCC samples in TCGA dataset into distinct molecular clusters. The optimal number of clustering was identified using the cumulative distribution function (CDF) curve [50]. Kaplan–Meier survival analysis was performed to estimate the prognosis of patients in each molecular cluster.

### Relationships between molecular clusters and clinical features, immune infiltration, and treatment response

Moreover, we also explored the distribution of clinical-pathological features among the distinct molecular clusters using a Chi-square test and one-way analysis of variance (ANOVA) using SPSS version 20.0 (SPSS Inc., Chicago, IL). To describe the differences of immune cell infiltration and the abundance of stromal cells in each distinct cluster, we implemented the R package, microenvironment cell populations-counter (MCP-counter) [51], single-sample gene set enrichment analysis (ssGSEA) [52], as well as the Estimation of Stromal and Immune cells in Malignant Tumors using Expression data (ESTIMATE) algorithm [53]. Meanwhile, we assessed the immunotherapeutic response of each cluster to immune checkpoint blockade (ICB) using the Tumor Immune Dysfunction and Exclusion (TIDE) algorithm [54]. In addition, IC50 concentrations were also assessed using the R package, ‘pRRophetic’, to measure the sensitivity of the ccRCC patients in each cluster to several chemotherapeutic agents in the GDSC database (Genomics of Drug Sensitivity in Cancer, https://www.cancerrxgene.org/).

### Establishment of a NK cell-related model

Based on the above molecular clusters, we performed differential gene expression (DGE) analysis on each couple of clusters using the R package, ‘limma’. The selection criteria were log fold change (FC) > 1 and adjusted *p*-value < 0.05. Then, we took the intersection between the significantly differential expressed NK cell-related genes (DE-NKRGs) of each cluster. The univariate Cox analysis was used to identify the DE-NKRGs with a threshold of *p* < 0.001. To construct the prognostic model, least absolute shrinkage and selection operator (LASSO) regression were performed on the TCGA database using the R package, ‘glmnet’. Based on the minimum criteria, the optimal tuning parameter λ and coefficients were calculated through tenfold cross-validation. The multivariable stepwise Cox regression model was further used to identify parameter values using the Akaike information criterion (AIC). Then, a prognostic gene model was constructed based on the linear combination of the LASSO regression coefficient (β) weighted by its mRNA expression level. The patients were divided into high-risk or low-risk groups based on the median score. Meanwhile, the ROC curve was constructed using the R package, ‘time ROC’ to estimate model performance. The Kaplan–Meier (KM) curve was analyzed using the R package, ‘survival’ to analyze the outcomes of both groups. Finally, we used the ICGC database to validate the prognostic value and robustness of the model.

### Relationships between risk score and clinical characteristics, immune infiltration, and therapeutic effects

The relationships between the risk score and multiple clinical factors, including neoadjuvant status, sex, and age, were estimated. Subsequently, we employed the MCP-counter method to compare the degree of immune cell infiltration between the high- and low-risk groups of the TCGA cohort. It is generally known that differences in the expression of immune checkpoint genes influence the response after immune checkpoint inhibitor treatment in malignant tumors. Therefore, we evaluated differences in the expression levels of the immune checkpoints downloaded from the HisgAtlas database (http://biokb.ncpsb.org/HisgAtlas/) between two risky groups [55]. The TIDE algorithm was further used to analyze the efficacy of immunotherapy. To explore the sensitivity to different types of treatments in each risk group of TCGA cohort, ridge regression was applied to assess the IC50 of the chemotherapy drugs.

### Predictive decision-tree model and nomogram construction

To explore the importance of the risk score and clinical factors, 4 clinical factors, age, gender, grade, and TNM stage, were extracted from the above analysis and used as input features, and were used along with the risk score to construct the decision tree model using R package, ‘rpart’. Univariate and multivariate Cox regression analyses were further performed to identify significant prognostic factors for ccRCC. Furthermore, the nomogram model, which integrated clinical features (age, gender, and tumor stage) and the risk score, was constructed using the R package, ‘rms’, to clearly and precisely predict the 1-, 3-, and 5-year outcomes of the patients [56]. Subsequently, the calibration curves were used to test the performance of the nomogram against the actual survival rate. Finally, time-dependent (tROC) analysis was performed using the R package, ‘survivalROC’ to estimate the predictive power of the nomogram, stage, grade, TNM stage, age, and risk score [57].

### Statistical analysis

Statistical analyses were performed using R version 4.0.4 and SPSS 26.0 software, while figures were drawn using GraphPad Prism 8.4.2 software. Differences between groups were compared using the Wilcoxon test or Kruskal–Wallis test. A *p* value < 0.05 was considered to indicate statistical significance in all analyses.

## Results

### Molecular clusters of ccRCC based on the NK cell-related genes

**Fig. 1:**
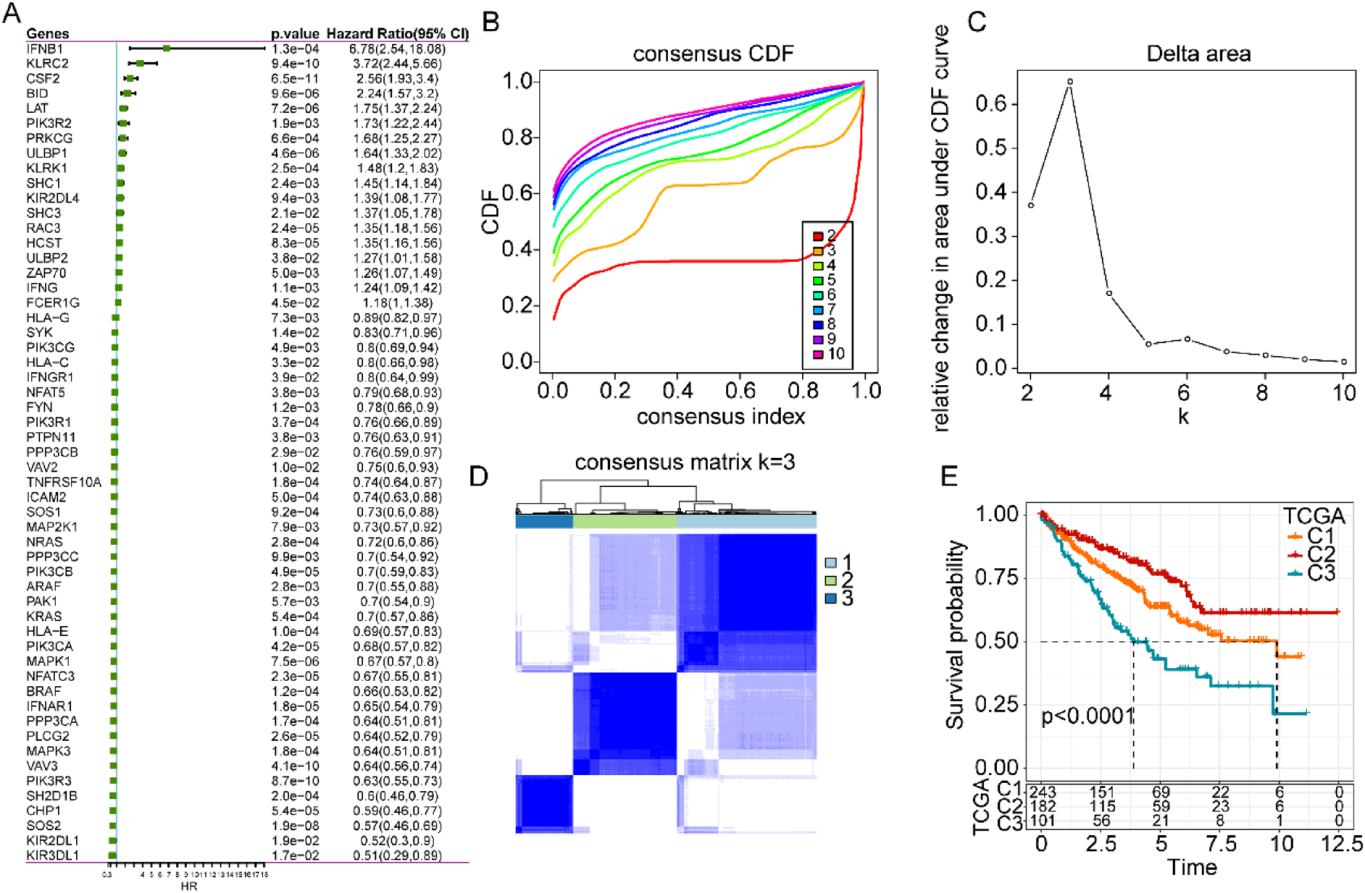
Development of molecular clusters based on the expression of NK cell-related gene in ccRCC using the TCGA database. **A** Univariate Cox analysis of 134 NK cell-related genes. **B** CDF curves for k ranging from 2 to 9. **C** Corresponding change in the area under the CDF curve. **D** Consensus clustering matrix for the optimal, *k* = 3. **E** Kaplan–Meier survival analysis of patients in cluster 1, cluster 2, and cluster 3

First, univariate Cox regression analysis was used to screen the NK cell-related genes that were significantly associated with the prognosis of ccRCC data obtained from the TCGA database. Overall, 55 genes were selected based on their prognostic value, including 18 hazardous genes and 37 protective genes (Fig. 1a). Based on the expression levels of these 55 genes, we conducted a consensus clustering analysis on the ccRCC patients to better understand the role of the NK cell-related genes in ccRCC. We continuously increased the clustering variable *k* from 2 to 9, and found that *k* = 3 produced the ideal cumulative distribution function (CDF) value and delta area (Fig. 1b, c). Therefore, the patients in the TCGA cohort were divided into 3 clusters of NK-related genes: C1, C2, and C3 (Fig. 1d). KM analysis illustrated that C2 genes increased overall survival (OS) and that the C3 patients had relatively poorer outcomes (Fig. 1e).

### Comparison of clinical features, immune characteristics of TME, and the therapeutic response between the 3 molecular clusters

The distribution of clinical features among the 3 clusters showed that differences exist based on the stage, grade, T stage, M stage, age, and gender of the 3 clusters in the TCGA database. Patients younger than 60 years with less advanced tumor stages were more concentrated in the C2 cluster (Additional file 1: Fig. S1A). Additionally, the interaction between the tumor immune microenvironment and cancer cells determined cancer progression and the efficacy of immunotherapy. The MCP-Counter algorithm was applied to identify the degree of infiltration of 10 immune cells and it was found that the immune scores were highest in the C2 cluster (Additional file 1: Fig. S1B). Through the ESTIMATE method, we also found significant differences in the infiltration of stromal and immune cells into tumor tissues. The results revealed that the C2 cluster possessed higher stromal and immune scores (Additional file 1: Fig. S1C). Based on these immune features, we determined the expression of immune checkpoint genes and most immune checkpoint genes were found to be highly expressed in the C2 cluster (Additional file 1: Fig. S1D). To further evaluate patient populations that may benefit from immunotherapy, the TIDE algorithm found that the C3 cluster had a higher TIDE score than the other 2 clusters (Additional file 1: Fig. S1E). The C2 cluster was found to be more sensitive to traditional chemotherapies, such as rapamycin, sunitinib, paclitaxel, sorafenib, crizotinib, and AKT inhibitor VIII (Additional file 1: Fig. S1F).

### Determination of crucial NK cell-related genes

**Fig. 2:**
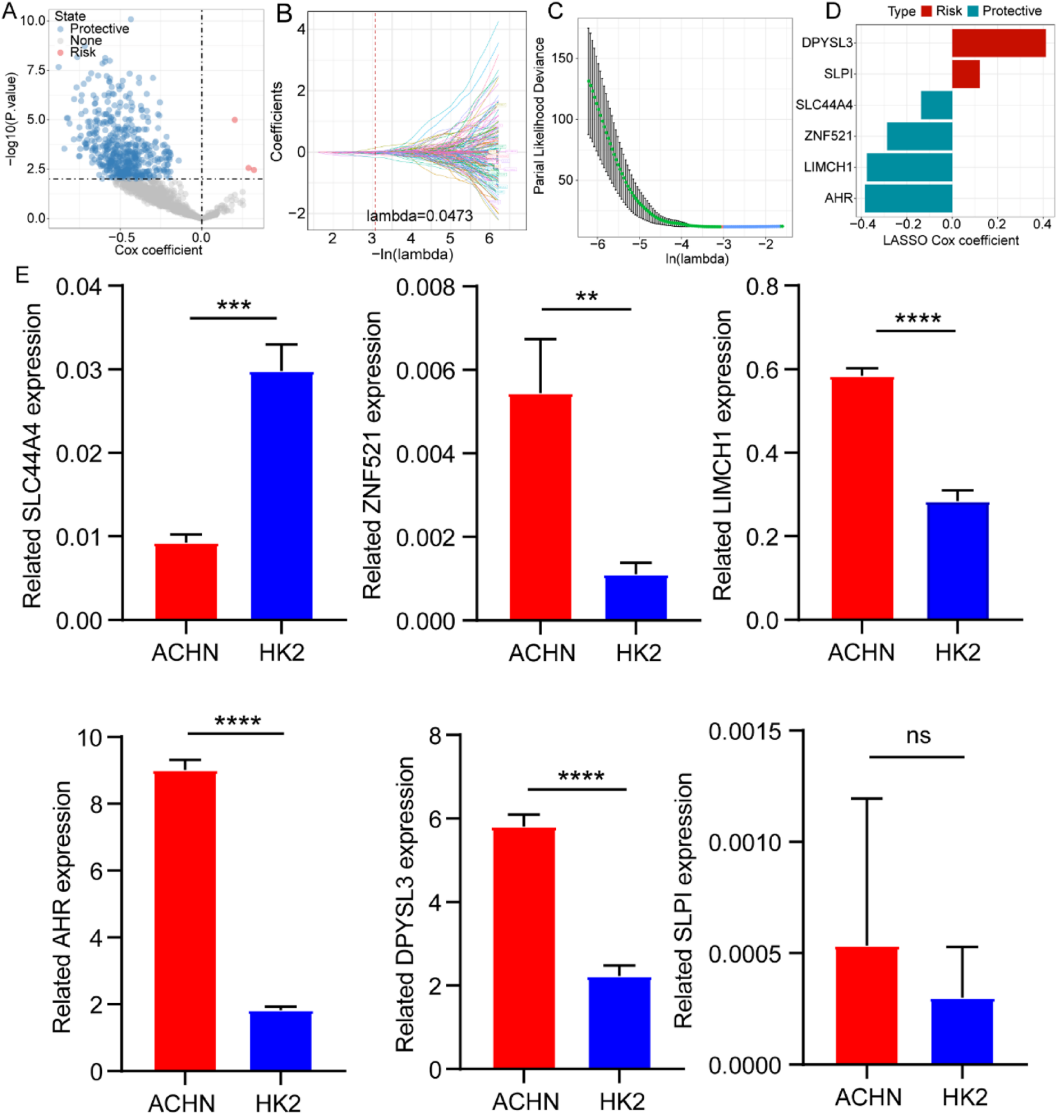
Construction and validation of a NK cell-related risk score model. **A** Univariate COX regression analysis of the DE-NKRGs in the TCGA database. **B** LASSO coefficient profiles. **C** Lambda selection through tenfold cross-validation. **D** Coefficient of the 6 screened genes. **E** Expression profiles of DPYSL3, SLPI, SLC44A4, ZNF521, LIMCH1, and AHR in the ACHN cells and HK2 cells.

We conducted differential gene expression analyses separately on each pair of clusters using the “limma” R package and obtained 1183 common differentially expressed NK-related genes (DE-NKRGs) for the intersection between the results. Furthermore, univariate COX regression analysis of the DE-NKRGs indicated that 518 genes were mainly involved in patient prognosis and included 3 risk-related genes and 515 protective genes (Fig. 2a). Then, the LASSO Cox regression was employed to shrink the scope of the genes and the model was found to be optimal when the value of lambda was 0. 0473 (Fig. 2b, c). The expression of 6 genes included 2 risk-related genes (DPYSL3 and SLPI) and 4 protective genes (SLC44A4, ZNF521, LIMCH1, and AHR) was estimated in two cell lines by qRT-PCR (Fig. 2d). The qRT-PCR results exhibited that the risk-related gene DPYSL3 was highly expressed and the protective gene SLC44A4 was lowly expressed in ACHN cells, which was consistent with expectations. Meanwhile, ZNF521, LIMCH1, and AHR expression were upregulated in the ACHN cells compared with the HK2 cells. The SLPI expression was no clear difference between ACHN and HK2 cells (Fig. 2e).

### Construction of a NK cell-related risk signature

**Fig. 3:**
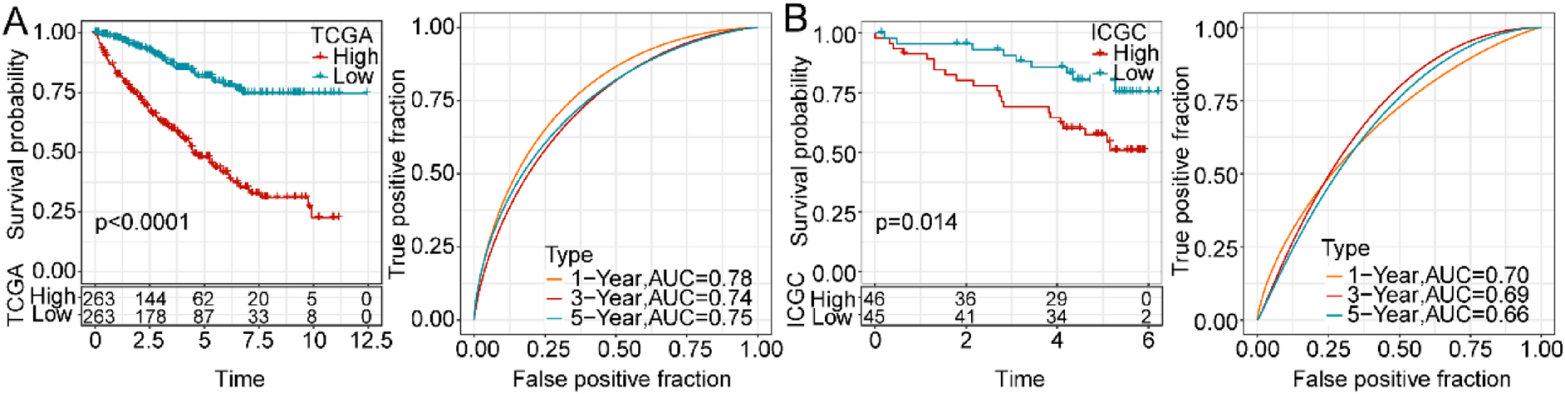
Exploration of the correlation of risk score with ccRCC prognosis. **A** Kaplan–Meier analysis and ROC curves at 1, 3, and 5 years of high- and low-risk groups in TCGA database. **B** Kaplan–Meier analysis and ROC curves at 1, 3, and 5 years of high- and low-risk groups in ICGC database

The selected 6 DE-NKRGs were incorporated into the risk score: risk score = − 0.375*LIMCH1-0.384*AHR-0.287*ZNF521 + 0.412*DPYSL3-0.137*SLC44A4 + 0.121*SLPI. Based on the median value, we separated the patients into low-risk and high-risk groups. It was observed that patients with a high-risk score had a poorer prognosis than that those with a low-risk score (Fig. 3a) [58]. The ROC analysis was conducted to analyze the prognostic efficiency of the risk score. The AUC of the TCGA cohort reached 0.78,95 after 1 year, 0.74,95 after 3 years, and 0.75,95 after 5 years. Similarly, the AUC of the ICGC cohort achieved 0.7,95 after 1 year, 0.69,95 after 3 years, and 0.66,95 after 5 years. Kaplan–Meier analyses of both TCGA and ICGC cohorts consistently showed that low-risk patients tended to have a more favorable outcome (Fig. 3b).

### Relationship between the risk score and clinical features, immune microenvironment, and treatment response

We further analyzed the distribution of the clinical features of patients in the TCGA cohort [59]. A remarkable difference exists between the 2 groups in terms of tumor stage. The C2 cluster showed a significantly lower risk score (Additional file 1: Fig. S2A). To elucidate the relationship between the risk score and the immune microenvironment of ccRCC, the MCP-counter tool was used to investigate differences in the infiltration of 10 immune cells between the high- and low-risk groups. The results highlighted that the low-risk group had a higher level of immune cell infiltration (Additional file 1: Fig. S2B) [60]. Moreover, we performed TIDE analysis to further explore the clinical utility of the risk score. The low-risk group possessed a lower TIDE score compared with the high-risk group (Additional file 1: Fig. S2C). In addition, patients in the low-risk group exhibited a higher degree of sensitivity to conventional drugs, such as rapamycin, sunitinib, sorafenib, crizotinib, and AKT inhibitor VIII (Additional file 1: Fig. S2D).

### Building a survival decision tree and a predictive nomogram to predict ccRCC survival

**Fig. 4:**
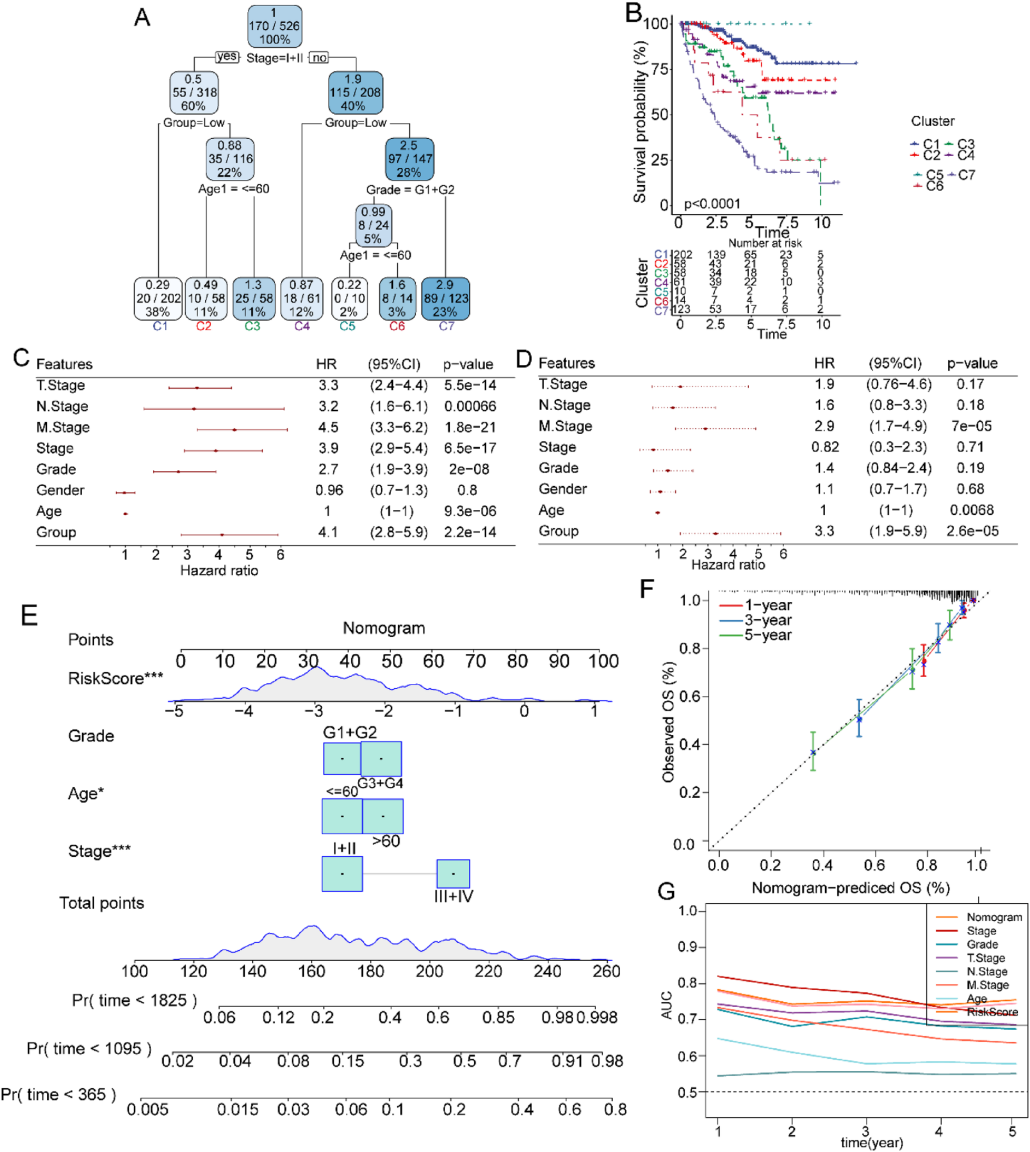
Establishment of a decision tree and nomogram by combining the risk score and clinical features of TCGA database. **A** Framework of a decision tree. **B** Survival analysis of the 7 clusters obtained using the decision tree. **C** Results of the univariate Cox analysis. **D** Results of the multivariate Cox analysis. **E** The nomogram constructed to predict the survival probabilities. **F** Calibration curves of the nomogram. **G** The tROC curves of the nomogram.

Additionally, validated the prognostic model using decision tree analysis integrating risk score and clinicopathological features, including age, gender, T stage, N stage, M stage, stage, and grade. Age, stage, grade, and risk score were entered as inputs into the survival decision tree to obtain 7 risk subgroups (Fig. 4a). Survival analyses also revealed statistically significant differences between the 7 risk subgroups (Fig. 4b). Next, we used univariate and multivariate Cox regression analyses and found that the risk score was the most significant factor for the prediction of ccRCC prognosis (Fig. 4c, d). To create a clinical approach for the prognostic estimation of ccRCC patients, the factors mentioned in the decision tree analysis were further applied to construct a nomogram (Fig. 4e). The results of the nomogram showed that the risk score had a dramatic influence on the survival prediction of ccRCC patients. The calibration plots for 1-year, 3-year, and 5-year survival probabilities showed high consistency between the results of the constructed nomogram and actual clinical conditions (Fig. 4f). Moreover, the tROC curves with high AUC values above 0.7 indicated that the nomogram and risk score provided an accurate prediction of ccRCC prognosis (Fig. 4g). These results demonstrate the favorable predictive value of our risk score.

## Discussion

Emerging evidence suggests that NK cells play essential and dual roles in diverse disease models such as infection, transplantation, autoimmunity, as well as cancer. Studies have reported that NK cells function as crucial components of tumor immunosurveillance, exerting a potent cytolytic effect by recognizing ligands presented on tumor cells and inducing the involvement of a series of NK activating receptors [61, 62]. For ccRCC, immune-based therapies (including targeted PD-1/PD-L1) have emerged as the main management modality since it has a restricted response to conventional chemotherapy [63–65]. Thus, a more comprehensive understanding of the features of immune cells of ccRCC patients is imperative for developing effective and improved immunotherapies [25].

Research has uncovered the heterogeneity of infiltrating NK cells in ccRCC, revealing their intricate association with tumor metastasis and differences in the efficacy of immunotherapy [66]. Sierra et al. observed an increased expression of PD-L1 on NK cells and a corresponding high infiltration inccRCC, which paradoxically correlated with worsen survival outcomes of ccRCC patients [67]. Ziblat et al. found that tumor-infiltrating NK cells (TINK) in ccRCC represent an activated residency phenotype, contributing to desensitizing NK cells to tumor cells and further limiting their anti-tumor effect [68].

To identify NK cell-related prognostic biomarkers for ccRCC, we initially identified three prognostic clusters based on NK cell-related gene expression from the TCGA and ICGC databases. Bioinformatics analysis of ccRCC revealed distinct clinicopathological characteristics, prognosis, immune infiltration properties, and therapeutic responses among these three NK cell-related clusters. Risk scores have been proven to allow clinicians to adopt more personalized clinical approaches and facilitate prognostic outcomes as high-throughput genomic technologies become increasingly integrated into clinical practice [69–72]. Zhang et al. structured a risk score model for oral squamous cell carcinoma based on the expression of the Shelterin complex gene (SG) to evaluate the prognosis and immunotherapy responses of patients [73]. Additionally, Cao et al. identified a risk model based on immune-related gene signatures to predict prognosis and guide individualized therapies for advanced RCC [74]. Considering the distinctive characteristics of the molecular clusters, we constructed a six-gene based NK-related risk score model to explore the association between NK-related genes and ccRCC development. Our research reported that the high-risk score group significantly implicated poorer outcomes, advanced clinical stages, reduced immune cell infiltration, and increased insensitivity to anti-tumor immune responses. Given its role in representing the clinical decision-making processes, we implemented a decision tree model to further validate the performance of risk scores. Importantly, we indicated that a proposed nomogram integrating risk scores with clinical characteristics showed superior effective predictive efficacy for ccRCC prognosis.

Recent advancements in imaging technology and genomics have significantly aided clinical physicians in stratifying risk, selecting treatment plans, developing follow-up strategies, and predicting patient outcomes for various diseases. The combination of radiomics features and genomic data has demonstrated promising applications in clinical settings, such as RCC [75, 76]. In this study, we primarily analyzed NK cell-related gene expression in ccRCC and established a corresponding prognostic model. We validated the model's robustness and efficacy by utilizing public ccRCC patient databases. Based on individual risk characteristics, the model can be utilized to stratify patients, predict treatment responses, and evaluate clinical prognoses. Further integration of our prognostic model with radiomics offers a meaningful direction for future research. By combining clinical radiological information of ccRCC patients, our model holds potential to provide more personalized risk assessment, elevated diagnostic accuracy, and refined treatment plans and follow-up strategies for patients.

However, our research has several limitations. First, the gene expression data used in our study were obtained from online platforms, and there is a lack of data from our own center to validate the constructed prognostic model. Second, we focused only on 6 genes with the most significant correlation with ccRCC prognosis to establish the model. Although we found evidence supporting the importance of the expression and prognosis of these 6 genes, their functional mechanisms during ccRCC development are worthy of further experimental studies and validation in vivo. The qRT-PCR merely confirmed the mRNA expression profiles of the 6 genes and further analysis is needed to expand the exploration of 6 genes at the protein level and also on the animal models.

## Conclusion

Taken together, we identified three distinct molecular clusters and proposed a generalized and robust prognostic model for ccRCC based on NK-related gene signatures. The risk score was confirmed to exert a powerful role for the prediction of ccRCC prognosis and can provide more appropriate guidance for the treatment of ccRCC patients.

### Supplementary Information

**Additional file 1. Fig. S1**: Relationship between molecular subtypes and clinical characteristics, immune cell infiltration, immune score, immune checkpoint genes, and treatment sensitivities. **A** Distribution of the clinical characteristics in the 3 molecular clusters. **B** Degree of infiltration of the 10 types of immune cells in the 3 molecular clusters. **C** Immune scores in the 3 molecular clusters. **D** Difference in the expression of immune checkpoint genes in the 3 molecular clusters. **E** Immunotherapy response in the 3 molecular clusters. **F** Sensitivities of traditional chemotherapy in the 3 molecular clusters. **Fig. S2**: Differences in clinical features, immune cell infiltration, immune escape and chemosensitivity between the high- and low-risk groups. Association between the NK cell-related Risk Score and **A** clinical features, **B** immune cell infiltration, **C** immune escape, and **D** chemosensitivity in the TCGA database. **Table S1**: The primer sequences for qRT-PCR.

## Data Availability

The data that support the findings of this study are available from the corresponding author upon reasonable request.
