# Supplementary material for: A promising natural killer cell-based model and a nomogram for the prognostic prediction of clear-cell renal cell carcinoma
Source: Eur J Med Res. 2024 Jan 24;29:73. doi: 10.1186/s40001-024-01659-0 (PMC10807100; doi:10.1186/s40001-024-01659-0)
Supplement: Supplementary file 1 — Additional file 1. Fig. S1: Relationship between molecular subtypes and clinical characteristics, immune cell infiltration, immune score, immune checkpoint genes, and treatment sensitivities. A Distribution of the clinical characteristics in the 3 molecular clusters. B Degree of infiltration of the 10 types of immune cells in the 3 molecular clusters. C Immune scores in the 3 molecular clusters. D Difference in the expression of immune checkpoint genes in the 3 molecular clusters. E Immunotherapy response in the 3 molecular clusters. F Sensitivities of traditional chemotherapy in the 3 molecular clusters. Fig. S2: Differences in clinical features, immune cell infiltration, immune escape and chemosensitivity between the high- and low-risk groups. Association between the NK cell-related Risk Score and A clinical features, B immune cell infiltration, C immune escape, and D chemosensitivity in the TCGA database. Table S1: The primer sequences for qRT-PCR. [file 40001_2024_1659_MOESM1_ESM.docx]

**Supplementary Figures**

**Fig. S1**

**
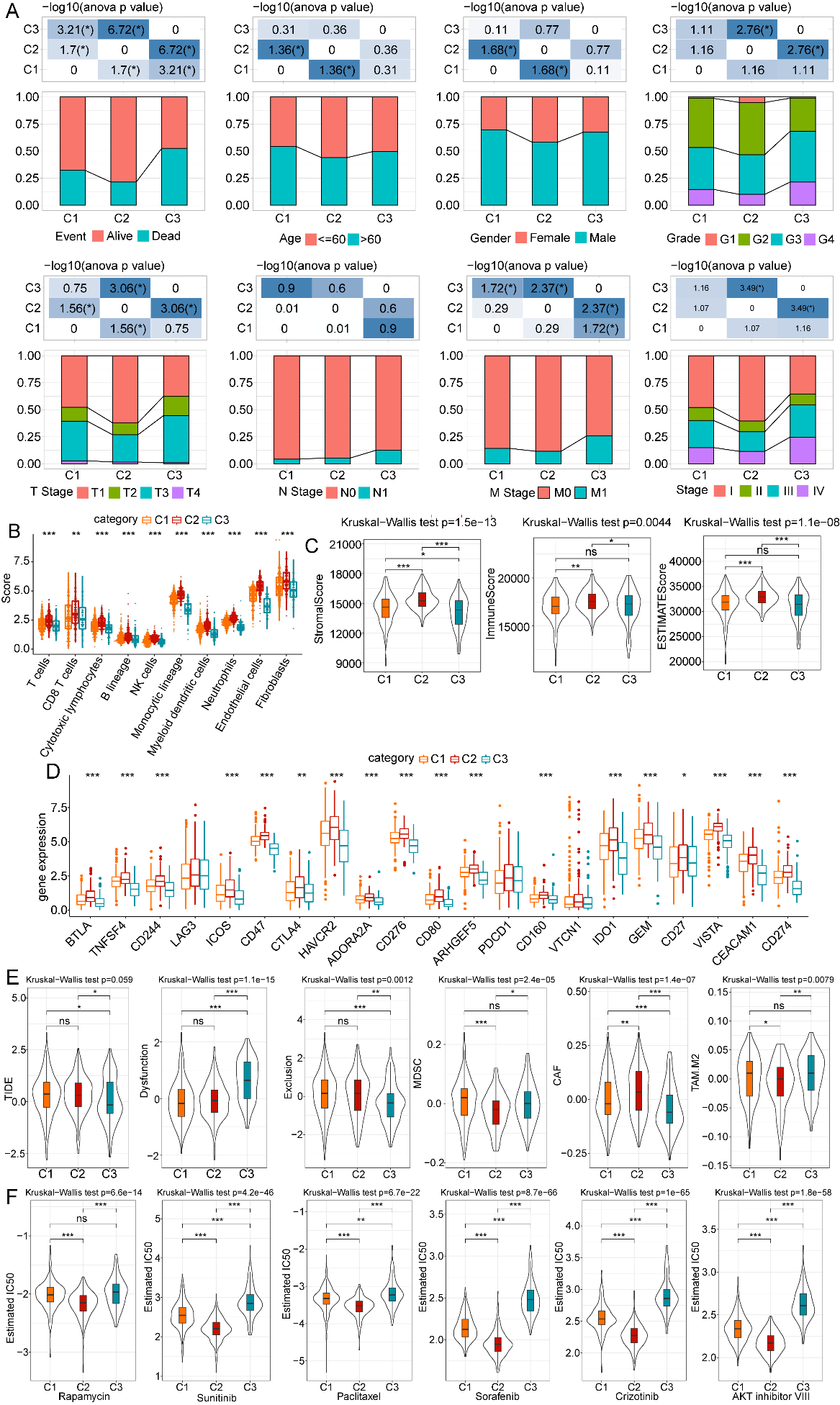
**

**Fig. S2**

**
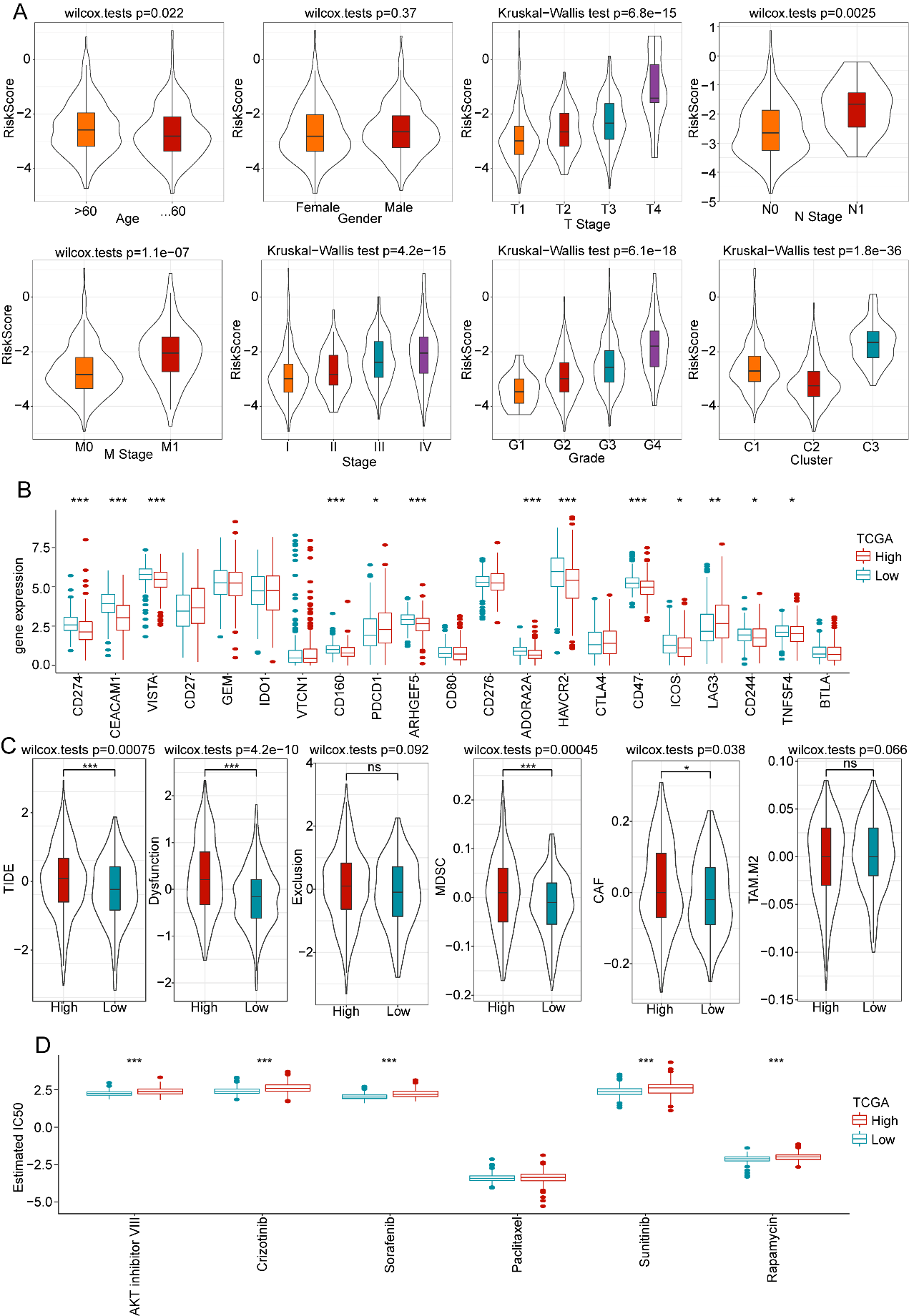
**

**Supplementary Figure legends**

**Fig. S1.** Relationship between molecular subtypes and clinical characteristics, immune cell infiltration, immune score, immune checkpoint genes, and treatment sensitivities.

(A) Distribution of the clinical characteristics in the 3 molecular clusters. (B) Degree of infiltration of the 10 types of immune cells in the 3 molecular clusters. (C) Immune scores in the 3 molecular clusters. (D) Difference in the expression of immune checkpoint genes in the 3 molecular clusters. (E) Immunotherapy response in the 3 molecular clusters. (F) Sensitivities of traditional chemotherapy in the 3 molecular clusters.

**Fig. S2.** Differences in clinical features, immune cell infiltration, immune escape and chemosensitivity between the high- and low-risk groups. Association between the NK cell-related Risk Score and (A) clinical features, (B) immune cell infiltration, (C) immune escape, and (D) chemosensitivity in the TCGA database.

| Table S1: The primer sequences for qRT-PCR. | | | | |
| --- | --- | --- | --- | --- |
|  | Sequence | |  |  |
| gene | Forward Primer | Reverse Primer | Length (bp) | Tm (℃) |
| AHR | ACATCACCTACGCCAGTCG | CGCTTGGAAGGATTTGACTTGA | 94 | 60 |
| DPYSL3 | CCTCGGCATAGATGGAACCC | CAGCAAGGAGTTGATGTAGTCC | 118 | 60 |
| LIMCH1 | CAGACGCCTTCACCAGATGTA | GATGAGGCAAGTCGGATTCAG | 79 | 59 |
| SLC44A4 | ATGCCCGAGACATCAGTGTTA | AACAGTAGGCTCAAGACCAGA | 83 | 61 |
| SLPI | CACCCCAAACCCAAGTAAGC | CATCACCCAGTTCCATCCCT | 89 | 59 |
| ZNF521 | AAGCAAGCGAAACCGAGATCC | GCCTCTTCTTACAATCTAGTGCC | 134 | 60 |
